# Malaria rapid diagnostic test (HRP2/pLDH) positivity, incidence, care accessibility and impact of community WASH Action programme in DR Congo: mixed method study involving 625 households

**DOI:** 10.1186/s12936-021-03647-9

**Published:** 2021-02-27

**Authors:** Nlandu Roger Ngatu, Basilua Andre Muzembo, Nattadech Choomplang, Sakiko Kanbara, Roger Wumba, Mitsunori Ikeda, Etongola Papy Mbelambela, Sifa Marie-Joelle Muchanga, Tomoko Suzuki, Koji Wada, Hasan Al Mahfuz, Tomohiko Sugishita, Hiroyuki Miyazaki, Shunya Ikeda, Tomohiro Hirao

**Affiliations:** 1 Department of Public Health, Kagawa University Graduate School of Medicine, Miki-cho, 761-0793 Japan; 2 Graduate School of Medicine, Dentistry and Pharmaceutical Sciences, Okayama University, Okayama, Japan; 3 Department of Public Health, School of Medicine, International University of Health and Welfare, Narita, Japan; 4 Faculty of Nursing, University of Kochi, Kochi, Japan; 5 Department of Tropical Medicine, Faculty of Medicine, University of Kinshasa, Kinshasa, Democratic Republic of Congo; 6 Department of Environmental Medicine, Kochi University Medical School, Nankoku, Japan; 7 National Center for Global Health and Medicine, Tokyo, Japan; 8 Department of International Affairs and Tropical Medicine, Tokyo Women’s Medical University, Tokyo, Japan; 9 Center for Spatial Information Science, University of Tokyo, Tokyo, Japan

**Keywords:** Democratic republic of congo, Household malaria, Incidence, Malaria care, Rapid diagnostic test

## Abstract

**Background:**

Malaria is one of the most prevalent and deadliest illnesses in sub-Saharan Africa. Despite recent gains made towards its control, many African countries still have endemic malaria transmission. This study aimed to assess malaria burden at household level in Kongo central province, Democratic Republic of Congo (DRC), and the impact of community participatory Water, Sanitation and Hygiene (WASH) Action programme.

**Methods:**

Mixed method research was conducted in two semi-rural towns, Mbanza-Ngungu (a WASH action site) and Kasangulu (a WASH control site) in DRC between 1 January 2017 through March 2018, involving 625 households (3,712 household members). Baseline and post-intervention malaria surveys were conducted with the use of World Bank/WHO Malaria Indicator Questionnaire. An action research consisting of a six-month study was carried out which comprised two interventions: a community participatory WASH action programme aiming at eliminating mosquito breeding areas in the residential environment and a community anti-malaria education campaign. The latter was implemented at both study sites. In addition, baseline and post-intervention malaria rapid diagnostic test (RDT) was performed among the respondents. Furthermore, a six-month hospital-based epidemiological study was conducted at selected referral hospitals at each site from 1 January through June 2017 to determine malaria trend.

**Results:**

Long-lasting insecticide-treated net (LLIN) was the most commonly used preventive measure (55%); 24% of households did not use any measures. Baseline malaria survey showed that 96% of respondents (heads of households) reported at least one episode occurring in the previous six months; of them only 66.5% received malaria care at a health setting. In the Action Research, mean incident household malaria cases decreased significantly at WASH action site (2.3 ± 2.2 cases vs. 1.2 ± 0.7 cases, respectively; p < 0.05), whereas it remained unchanged at the Control site. Similar findings were observed with RDT results. Data collected from referral hospitals showed high malaria incidence rate, 67.4%. Low household income (ORa = 2.37; 95%CI: 1.05–3.12; p < 0.05), proximity to high risk area for malaria (ORa = 5.13; 95%CI: 2–29-8.07; p < 0.001), poor WASH (ORa = 4.10; 95%CI: 2.11–7.08; p < 0.001) were predictors of household malaria.

**Conclusion:**

This research showed high prevalence of positive malaria RDT among the responders and high household malaria incidence, which were reduced by a 6-month WASH intervention. DRC government should scale up malaria control strategy by integrating efficient indoor and outdoor preventive measures and improve malaria care accessibility.

## Background

Worldwide, progress has been made towards malaria control and elimination in the two decades. However, many countries of the sub-Saharan Africa (SSA) still have endemic malaria transmission. Thus, the search for improved malaria control or elimination strategies is still in progress [1]. Malaria is transmitted by the *Anopheles* mosquito, and it is one of the most prevalent and deadliest illnesses in developing countries [2, 3]. The World Health Organization (WHO) estimates that the disease has caused between 473,000 and 789,000 deaths in the world in 2012, which was lower compared to 1, 000,000 deaths per year reported in the 1990s [2]. Estimates show that 90% of malaria-related deaths in the world occur in SSA, 40% of them occurring in the Democratic Republic of the Congo (DRC) and Nigeria. The high prevalence of asymptomatic malaria among young women and men is an important contributor in high transmission areas. In addition, in SSA, households lose approximately 25% of their total income to malaria-related expenses, suggesting that malaria is a disease that causes or deepens poverty [3–5].

Recently, the WHO reports have shown progress made by several countries in reducing malaria burden, and estimated showed that more than 6,000,000 malaria deaths have been averted in SSA between 2000 and 2015 [6]. Nevertheless, the emergence of drug-resistant malaria parasites and pesticide-resistant *Anopheles* mosquitoes, inaccessibility to treatment for most at risk population, residual and outdoor transmission, the absence of rigorous evaluation of the effectiveness of malaria interventions and the absence of malaria vaccine are among factors that hinder malaria control and elimination programmes in most endemic countries [7–9]. Despite a noticeable reduction in global malaria burden in the last two decades, recent trends show stagnation of the progress made and an increase of disease burden in some countries. Additionally, nowadays malaria experts agree that to achieve malaria eradication, interventions should focus not only on the disease prevention, but also on reducing the disease transmission [10, 11].

In fact, most of malaria preventive measures implemented to scale up malaria control are those applied indoor. However, other contributing factors to the disease transmission, such as outdoor environmental factors, which increase mosquito population in residential area, are often excluded when designing malaria control interventions. In DRC, apart from challenges related to the availability of and use of malaria preventive measures, issues related to accessibility to health care services, including malaria care is another challenge [12]. A previous pilot study conducted in Congolese rural and urban counties showed that poor WASH and low income were associated with malaria [13]. Furthermore, other previous works showed evidence on the effects of malaria on workers’ absenteeism, productivity, medical costs [14, 15], population growth, as well as children developmental retardation and premature death [16, 17].

Currently, there is a growing concern about the widespread anti-malarial drug resistance in *Plasmodium* parasites, and the disease vector’s resistance to most commonly used insecticides in bed nets, which contribute to the failure of malaria prevention programmes in most endemic countries such as DRC. In DRC, malaria vector control policy is based on the use of long-lasting insecticidal bed nets (LLINs) [18, 19]. A recent study that assessed gene mutations involved in resistance phenomenon to pyrethroid and dichlorodiphenyl-trichloroethane (DDT)—two of most used insecticides against the disease vector—showed that 85% of *Anopheles gambiae* collected in DRC carried the *kdr* mutations [20]. This, suggests the necessity to find novel approaches susceptible to help scaling up malaria control programme in the Congo.

Moreover, despite the United Nations’ resolutions calling for accelerating progress towards equitable access to health services some countries of the SSA region, DRC in particular, are still left behind in terms of ensuring primary health care access to populations. There have been no studies conducted in central Africa region (in DRC in particular) that explored the impact of WASH intervention coupled with anti-malarial education at community level. The present research assessed the spatial malaria risk distribution, disease incidence and evaluated the impact of a community participatory WASH action on household malaria incidence in Kongo Central province, DRC. Additionally, this study searched to determine malaria trend in health settings located at the study sites using hospital-based epidemiological data.

## Methods

### Study design and sites

**Fig. 1:**
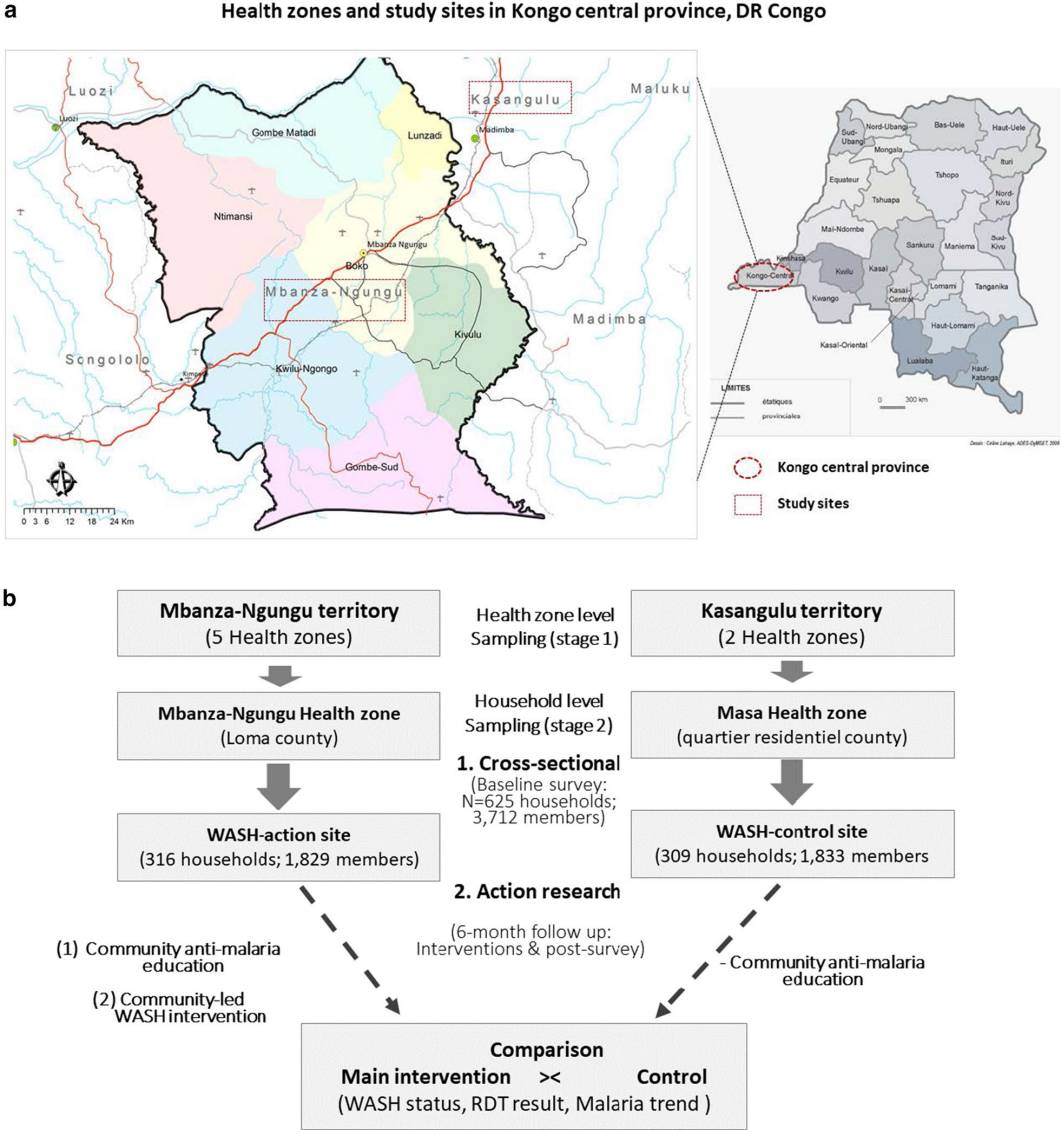
Maps of DR Congo (**a**) showing the study area in Kongo central province and main study flow chart (**b**) comprising both study designs of the main study ( Source of original maps: Celine Lahaye, ADES-DyMSET (2006) and CAID – DR Congo (Cellule d’ Analyse des Indicateurs de Developpement), 2017). RDT, rapid diagnostic test for malaria; WASH, water, sanitation and hygiene

Mixed method research comprising three distinct studies was conducted in two rural towns of Kongo Central province, DRC, from 1 January 2017 through March 2018. The main study, which involved 625 households (3,712 household members), consisted of an Action Research in which pre-(baseline) intervention and post-intervention surveys were carried out using the Malaria Indicator Questionnaire from the World Bank and WHO Malaria Programme in Africa and Madagascar [13, 21]. It was a 6-month prospective study comprising two interventions: community participatory WASH action and malaria education campaign. The latter intervention was implemented in both study sites (Fig. 1a). In addition, baseline and end-of-study malaria testing was performed among the survey responders (household heads).

The second study was a prospective hospital-based epidemiological study was conducted from 1 January through June 2017; medical records of patients were collected during the 6-month period, which was conducted using medical records of patients admitted from 1 January through 30 June 2017 at two randomly selected referral health settings.

### Sampling procedure and inclusion criteria

In the main study, a two-stage cluster sampling technique was used to randomly select two study sites, at the ‘health zone’ and municipality (county) levels as shown in Fig. 1b. Loma county (WASH action site) was randomly selected in Mbanza-Ngungu health zone in the rural town of Mbanza-Ngungu, located at 154 km from the capital Kinshasa, with an area of 8,460 km^2^. It has a population of 651,092. On the other hand, Quartier residentiel county (Control site) was randomly selected in Kasangulu health zone in the town of Kasangulu. Located at 33 km from the capital Kinshasa, Kasangulu has an area of 4,680 km^2^ and a population of 194,190 inhabitants. In DRC, a health zone consists of primary operational units of the health system and, usually, it covers a population of 100,000–150,000 inhabitants in rural areas and 200,000–250,000 inhabitants in an urban area [22]. The following criteria were used to select the county where the study should be conducted: (1) having a referral health setting under the supervision of the health zone inspector, and (2) the health setting should have a well-handled patients’ records. Hospital-based epidemiological data were collected at each study site to determine malaria incidence.

In the main study, all households from the randomly selected study sites having at least three members were eligible. Considering a power of 80% (β = 0.80) for α value of 0.05, we expected to have at least 200 households participate in this research. Within each study site, blocks of 50 houses were created; thereafter, data collectors have randomly selected every second house on each street in the area of each block. Data collectors were public health nurses and doctors who served under the supervision of one of the authors (WR) who is professor at the Faculty of Medicine, University of Kinshasa in DRC.

Additionally, only households having at least three members were finally included in the study. In total, 625 households (3,712 individuals) were surveyed, including 316 (50.6%) from the WASH action site and 309 (49.4%) from the Control site.

### Surveys and interventions

Surveys were conducted simultaneously at both study sites at baseline and at the end of six-month intervention period, following a schedule that was announced to residents by local health zone staff. The French version of malaria indicator survey (MIS) questionnaire was used in this study. It is a validated questionnaire used by the National Malaria Programme of several French speaking African countries to estimate household malaria burden.

MIS comprises an informed consent form and questions related sociodemographic and anthropometric characteristics, household characteristics, past medical history, WASH status, malaria preventive measures, malaria status and care. All household heads participated in the baseline and post-intervention surveys. Additionally, home visits were undertaken by local collaborative research team and Health Zone staff to evaluate WASH status at home and in the living environment, and check indoor and outdoor preventive measures used by household members, and ascertain consistency of their use. A hand-held GPS GIS device was used to collect data on geospatial localization of mosquito breeding sites; that is to estimate the distance between residences and mosquito breeding sites (grassy area, stagnant water spot, garbage spot and river side). We assumed that when a residence was located at less than 200 m from a mosquito breeding site, household members were considered at high risk for malaria.

Anti-malaria interventions comprised the following actions: (1) community WASH action consisting of a weekly participatory hygiene and sanitation transformation (PHAST) was carried out only in WASH action site in order to clean the residential environment and eliminate mosquito breeding spots; (2) community anti-malaria education. The latter was implemented in both study sites after the baseline survey. Education sessions were organized in communities, schools and leaflets that display risk factors and behaviors were being distributed to the participants as well as household heads in each study site. The PHAST approach is a learning methodology commonly used to prevent a broad range of infectious diseases at community level, through improvement of hygiene behaviors and sanitation, and encourages a better community management of water and sanitation services. In this study, PHAST was extended to periodic cleansing of the living environment, by local volunteers and community members.

### Diagnostic procedure for malaria and geospatial categorization of high risk area

Participants underwent the rapid diagnostic test (RDT) for malaria at baseline and at the end of the study. Blood sample was collected via finger prick into Heparin-coated tube. RDT is a validated diagnostic test for malaria; it has high accuracy for malaria diagnosis; it has the advantages of rapid-detection and it is easy to use and cost-effective. Thus, it is useful diagnostic procedure in resource-limited and endemic areas for malaria, particularly. It can detect malaria parasite’s specific antigens in the blood: histidine-rich protein-2 (HRP2) and lactate dehydrogenase (LDH). The test allows to diagnose malaria caused by *Plasmodium falciparum* and other *Plasmodium* species [23]. All participants with a positive RDT at baseline received malaria treatment and were followed for six months.

Regarding the hospital-based epidemiological study, only patients admitted to internal medicine and pediatric departments o between 1 January through 30 June 2017, and whose records showed final diagnoses were included; records that showed no diagnosis were excluded. For medical records showing comorbidities, the first diagnosis was considered.

In general, measures that reduce outdoor and indoor mosquito population are believed to play a crucial role in reducing malaria prevalence, especially in malaria endemic countries. Thus, during WASH intervention, mosquito breeding sites, which are considered high risk areas, were targeted.

Households living in proximity (distance less than 200 m) to a river, still and stagnant water/grassy spots were considered high risk areas for malaria. On the other hand, residences located at higher altitude or far from river, grassy/stagnant water spots were at lower risk for malaria.

### Outcome variables and statistical analysis

Outcome variables were the following: (1) prevalence of positive RDT among responders, (2) survey-based self-reported household incident malaria (number of doctor-diagnosed malaria cases in household members at any health setting), and (3) hospital-based malaria incidence from data registered at selected referral hospital at each study site. Data are presented as proportions for categorical variables, whereas means and their standard deviations are used for continuous variables. Comparisons within and between study groups were performed using paired t test (for incident malaria incidence cases) and chi-square test (for categorical variables); however, for categorical variables with repeated measures (RDT), McNemar’s test was used. All variables that showed a significant or marginally significant association with household malaria in the bivariate logistic regression analysis were subjected to a multivariate analysis model to determine predictors of malaria. Analyses were performed with the use of Stata software version 15.

## Results

### Baseline characteristics of respondents and households

Of the 625 respondents (household heads), 70.9% were females; overall mean age was 33.8 ± 8.8 years. The majority (66.2%) of the respondents were married men and women. Regarding education level, most respondents (64.6%) had high school level, followed by those with primary education level (13.6%), whereas 10.2% were illiterate. Mean household size was 5.9 ± 2.8 members, and almost 80% of them earned less than 100 US dollars a month. About 25% of households used appropriate/acceptable latrines. Baseline survey results also revealed that periodic sanitation intervention was not carried out in the residential area; this was supported by 97.6% of the respondents; 18.6% reported that the general sanitation status in their living environment was unacceptable (11.3% at control site vs. 25.6% at WASH action site) (Table 1).

**Table 1:** Characteristics of the respondents

Characteristics of respondents			
	Control site	WASH action site	All respondents
	n = 309 (%)	n = 316 (%)	N = 625 (%)
Gender (%)			
Male	71 (23)	111 (35.1)	182 (29)
Female	238 (77) ^*^	205 (64.9) ^*^	444 (71)
Age (years; mean ± SD)			
24–50	33.71 ± 8.74	33.86 ± 8.48	33.73 ± 8.68
51–98	60.17 ± 8.51	62.73 ± 9.25	61.27 ± 8.88
Marita status			
Married	205 (66.3)	209 (66.1)	414 (66.2)
Divorced	22 (7.1)	21 (6.7)	43 (6.9)
Widower	32 (10.4)	15 (4.7)	47 (7.5)
Single with child/children	28 (9.1)	37 (11.7)	65 (10.4)
Single without child	22 (7.1)	34 (10.8)	56 (8.9)
Education			
No education	19 (6.1)	45 (14.2)	64 (10.2)
Primary	37 (12)	48 (15.2)	85 (13.6)
High school	203 (65.7)	201 (65)	404 (64.6)
College/university	40 (12.9)	14 (4.5)	54 (8.6)
Technical/professional school	10 (3.2)	8 (2.5)	18 (3)
Smoking status			
Never smoked	306 (99)	284 (89.9)	590 (94.4)
Has quit (> 36 months)	0 (0.0)	15 (4.7)	15 (2.4)
Yes	3 (1)	17 (5.4)	20 (3.2)
Alcohol consumption			
Never	257 (83.1) ^*^	207 (65.5)	464 (74.2)
Has quit (over 36 months)		15 (4.7)	22 (3.5)
At most 2 glasses/day	7 (2.3)	53 (16.8)	73 (11.7)
More than 2 glasses/day	20 (6.5) 25 (8.1)	41 (13)	66(10.6)
Physical activity (≥ 20 min 2x/week) No	209 (67.6)	105 (33.2)	316 (50.6)
Yes	100 (32.4)	211 (66.8)	309 (49.41)

Characteristics of the households			
Household characteristics	Control site (Kasangulu)	WASH action site (Mbanza-Ngungu)	All households
	n = 309 (%)	N = 316 (%)	N = 625 (%)
*Sociodemographic characteristics*			
Household/family size			
1–5	126 (40.8)	152 (48.1)	278 (44.5)
6–10	153 (49.5)	148 (46.8)	301 (48.2)
≥ 11	30 (9.7)	16 (5.1)	46 (7.4)
Monthly income (US$) < 100	221 (44.38)	277 (55.62)	498 (79.68)
100 or higher	79 (62.20)	48 (37.80)	127 (20.32)
WASH status/ cleanliness of residential environment			
Latrine type (n = 603)			
Appropriate with flushing system	8 (2.6)	5 (1.6)	13 (2.1)
Appropriate with manual watering system	47 (15.2)	99 (31.3)	146 (23)
Inappropriate but covered	68 (22)	54 (17.1)	122 (19.5)122 (19.5)
Inappropriate and uncovered	177 (57.3)	126 (39.9)	303 (48.5)
No latrine	5 (1.6)	14 (4.4)	19 (3)
Other/ no answer	4 (1.3)	11 (3.5)	22 (3.5)
Shared latrine? (n = 625)			
Yes	49 (22.69)	167 (77.31) ^*^	216 (34.56)
No	260 (63.73)	149 (36.43)	409 (65.44)
Ecological feature of residence area			
Proximity to river	42 (13.6)	17 (5.4)	59 (9.4)
Proximity to/residence on mountain/hill	20 (6.5)	4 (1.3)	24 (3.8)
Proximity to garden, grass/bush	66 (21.3)	88 (27.8)	154 (24.6)
Proximity—stagnant water spot	54 (17.5)	92 (29.1)	146 (23.4)
Proximity or residence in slum	20 (6.5)	15 (4.7)	35 (5.6)
Proximity to river and mountain	38 (12.3)	4 (1.3)	42 (6.7)
Proximity—river and slum	49 (15.8)	20 (6.3)	69 (11)
Nothing particular	11 (3.6)	64 (20.2)	75 (12)
Other/no answer	9 (2.9)	12 (3.8)	21 (3.4)
Periodic cleansing in residential area			
Yes	5 (11.6)	10 (3.2)	15 (2.4)
No	304 (98.4)	296 (95.8)	610 (97.6)
Other/no answer	0 (0)	3 (1)	0 (0)
Cleanliness of residential area			
Good	135 (43.7)	13 (4.1)	148 (23.6)
Acceptable	139 (45)	222 (70.3)	361 (57.8)
Not acceptable	35 (11.3)	81 (25.6)	116 (18.6)
Other/no answer	0 (0)	0 (0)	0 (0)
Frequency of mosquito bites			
High frequency	86 (27.8)	134 (42.4)^*^	220 (35.2)
Moderate	184 (59.5)	153 (48.4)	337 (53.9)
No bite or rare	38 (12.3)	29 (9.2)	67 (10.7)
Other/no answer	1 (0.3)	0 (0)	1 (0.2)

*p value less than 0.05

### Malaria preventive measures (baseline survey) and trend of prevalence of positive RDT

**Fig. 2:**
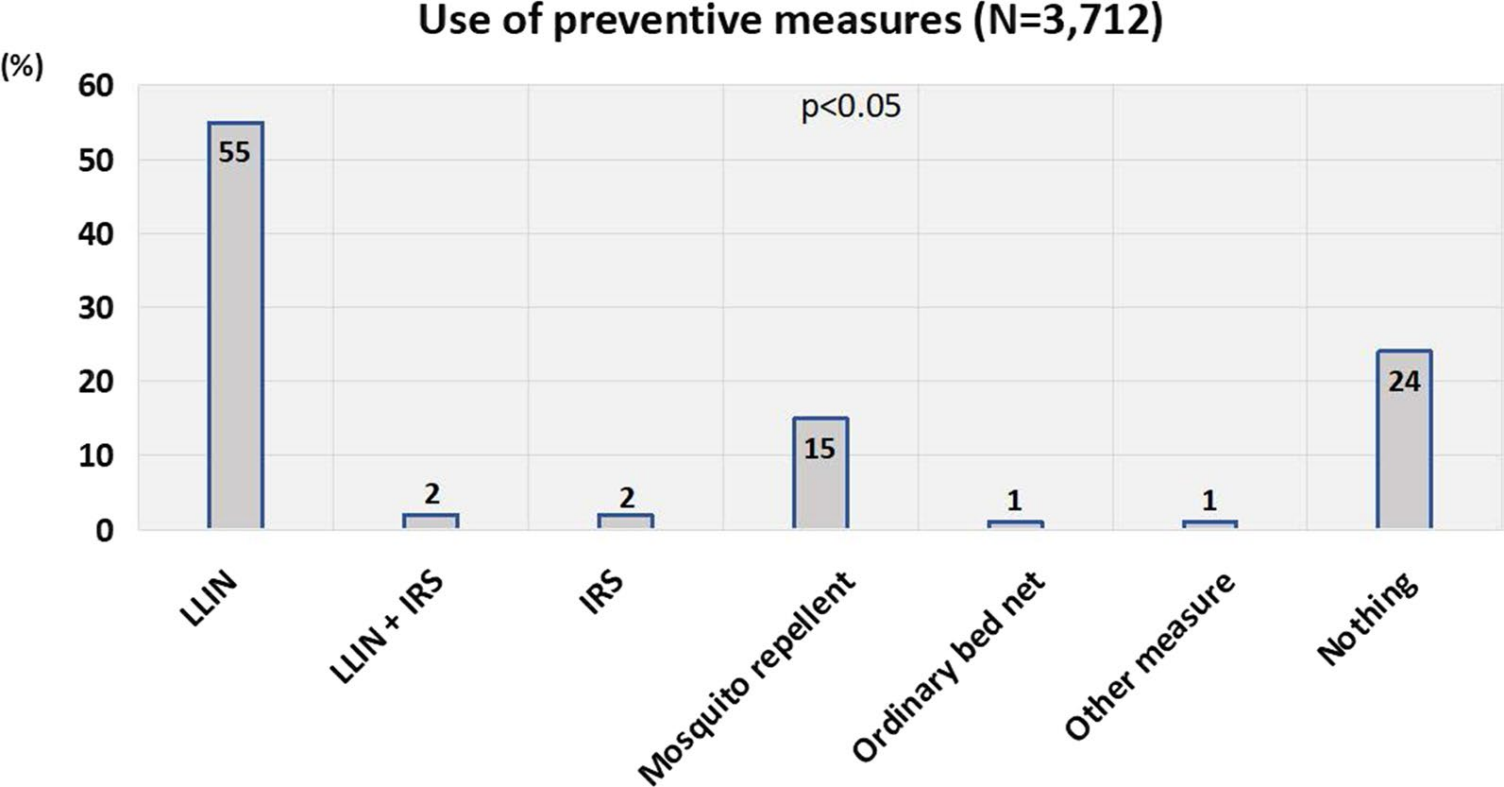
Malaria preventive measures used by household members. IRS, indoor residual spraying; LLIN, long-lasting insecticide treated net. The figure shows that LLIN was the most used anti-malaria measure (55%) in households followed by mosquito repellent (15%), whereas 24% of households did not use any preventive measure (p < 0.05)

Long-lasting insecticide-treated net (LLIN) was the most commonly used malaria preventive measure (55% of households), followed by mosquito repellent (15%), whereas indoor residual spraying (IRS) and the combination of LLIN and IRS accounted for 2% each. Strikingly, 24% of households did not use any of the measures as shown in Fig. 2.

**Fig. 3:**
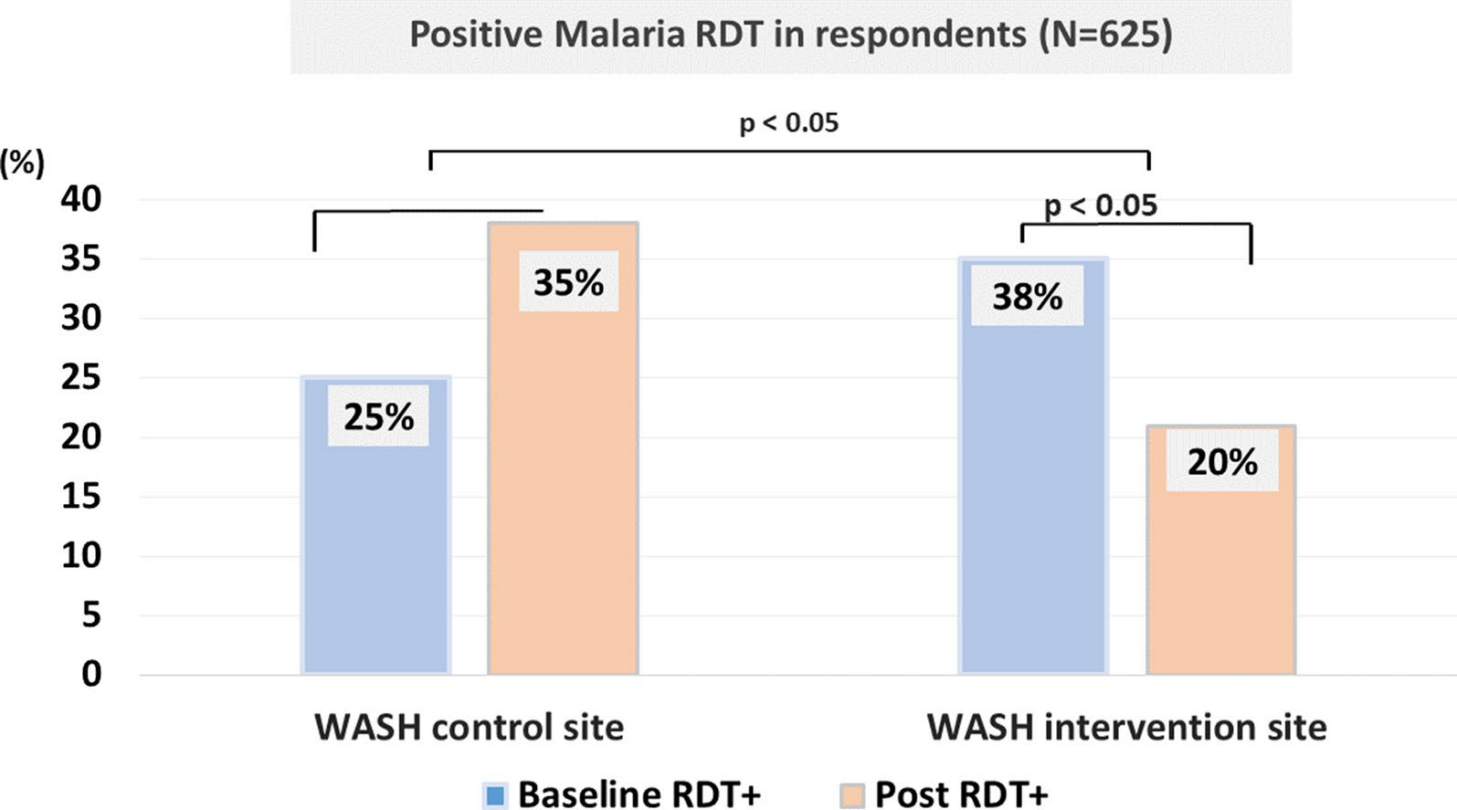
Prevalence of positive RDT for malaria among the respondents (household heads). NS, not significant; RDT + , rapid diagnostic test for malaria; WASH, water, sanitation and hygiene. The figure shows a significant decrease of prevalence rate of positive RDT at WASH action site (38% *vs*. 20%; p < 0.05) as compared to baseline RDT result

Regarding malaria testing among the respondents, results showed that the prevalence of positive RDT increased in the post-intervention RDT at the Control site, but not significantly (25% in pre-test vs. 35% in post-test, p > 0.05), whereas a marked reduction was observed in the WASH-action site (38% vs. 20%, respectively; p < 0.05) (Fig. 3).

### Survey-based self-reported incident malaria (at least one episode) and care accessibility

**Fig. 4:**
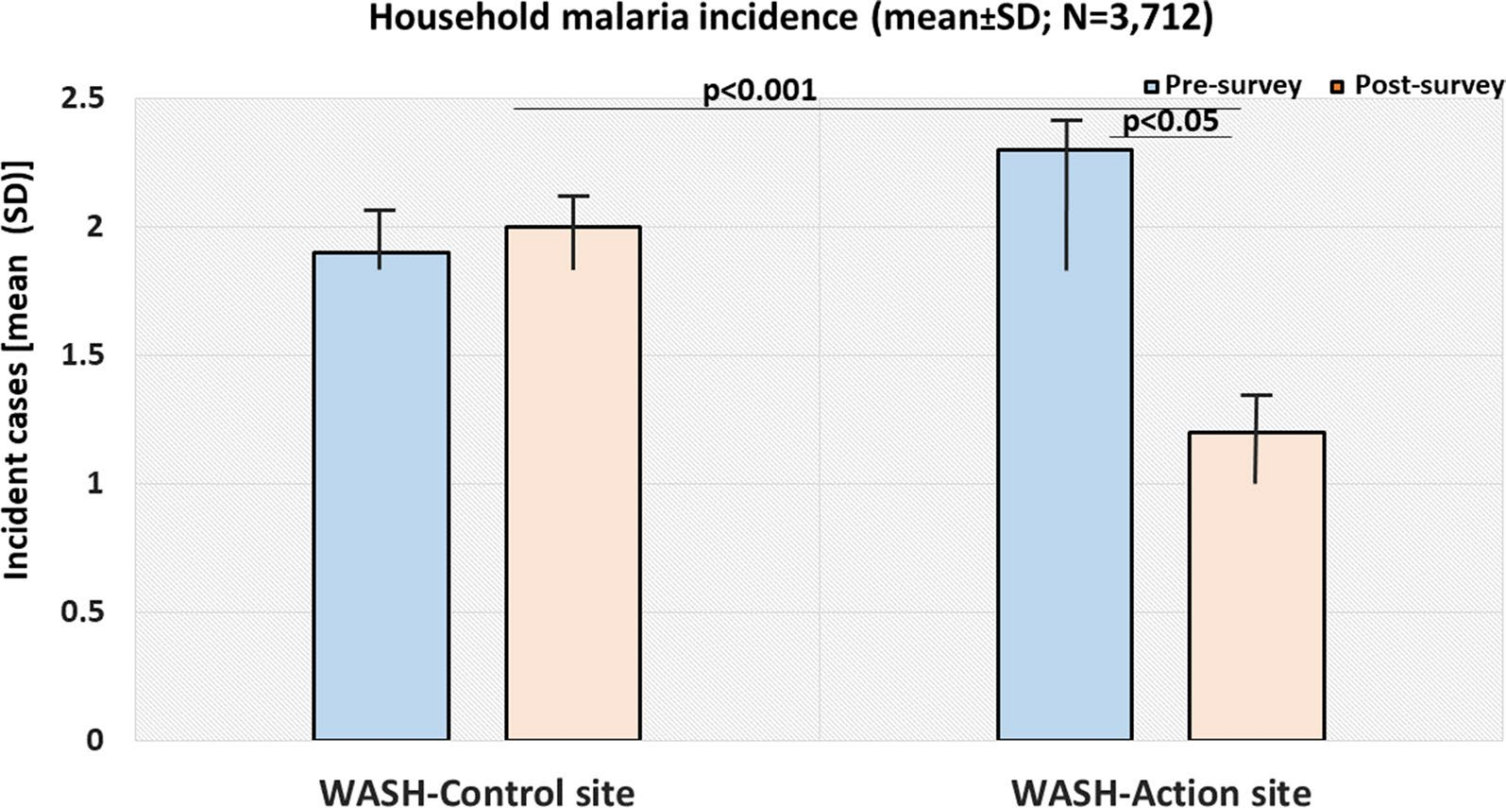
Survey-based self-reported incident household malaria before (baseline) and after interventions. SD, standard deviation; p, p-value. The figure shows a significant decrease in incident malaria cases (mean values) among household members at WASH action site following interventions (p < 0.05)

When comparing the malaria status before and after intervention, the disease incidence decreased among the respondents (96% *vs.* 52%, respectively) (not shown). Of the 96% of respondents who reported malaria event at baseline, only 66.5% received malaria care at a health setting. At household level, mean incident malaria cases decreased markedly in the WASH action site (2.3 ± 2.2 cases in pre-test vs. 1.2 ± 0.7 in post-test; p < 0.05) following 6-month sanitation intervention; however, no significant change was observed at the Control site (1.8 ± 0.5 vs. 2.0 ± 0.7, respectively) (Fig. 4).

### Survey-based self-reported incident household malaria according to residential area (baseline data)

**Fig. 5:**
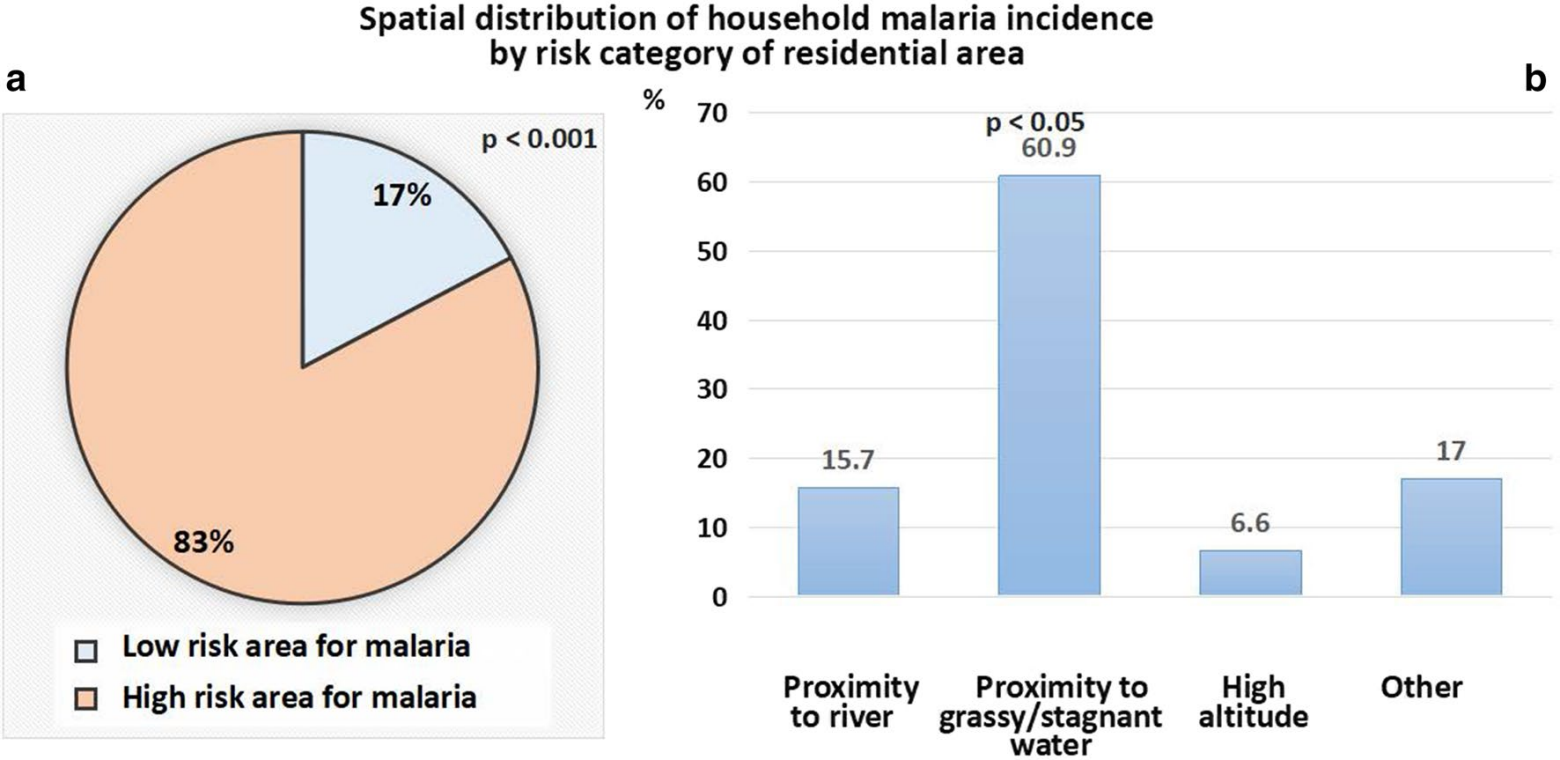
Spatial distribution of household incident malaria by risk category of residential area (**a**) and proximity of risk geographical high malaria risk area (**b**) (GIS-based data). The figure shows that higher malaria incidence was found among households living in proximity to high malaria risk areas such as grassy areas/stagnant water (60.9%) and residences near river (15.7%)

Each study site was divided into areas of low and high risk for malaria as described in methods section. Results showed that household malaria incidence rate was markedly higher (83%) among households with residences located at high risk areas as compared to those living in low risk areas (83 vs. 17%, respectively; p < 0.001). Moreover, household malaria incidence rate was highest (60.9%) among households living in proximity to grassy and/or still and stagnant water spots (Fig. 5a, b).

### Predictors of household malaria (baseline survey data)

The multivariate logistic regression analysis (with adjustment for age, gender and occupation) showed that household malaria was positively associated with household size (ORa = 1.39; 95%CI: 2.62–4.89; p < 0.05), proximity of residence to high risk area for malaria (ORa = 5.13; 95%CI: 2.29–8.07; p < 0.001) and the frequency of mosquito bites (2.68; 95%CI: 2.84–4.17; p < 0.05), whereas inverse association was found between household malaria and income status (OR = 2.37; 95%CI: 1.05–3.12; p < 0.05), also between household malaria and general WASH status in the residential environment (ORa = 4.10; 95%CI:2.11–7.08; p < 0.001) (Table 2).

**Table 2:** Predictors of household malaria (multi-variate analysis)

Predictors	ORa (SD)	95% CI	p-value
Age (< 40 years vs. 40 or older)	1.03 (0.02)	0.98–1.07	0.184
Gender (M vs. F)	0.27 (0.21)	0.05–1.27	0.099
Household size (2–5 vs. > 5)	1.39 (0.16)	2.62–4.89	< 0.05
Education level (Low vs. high)	0.88 (0.27)	0.48–1.62	0.701
Living environment (high risk vs. low risk)	5.13 (0.9)	2.29–8.07	< 0.001
Income status (US$) (< 100 vs. 100 or higher)	2.37 (1.42)	1.05–3.12	< 0.05
General WASH status (poor vs. good)	4.1 (0.5)	2.11–7.08	< 0.001
Mosquito bite (frequent vs. less frequent)	2.68 (0.27)	2.84–4.17	< 0.05

Multiple logistic regression model was performed with adjustment for age, gender, occupation

### Hospital-based malaria incidence (epidemiological data from referral hospitals)

**Fig. 6:**
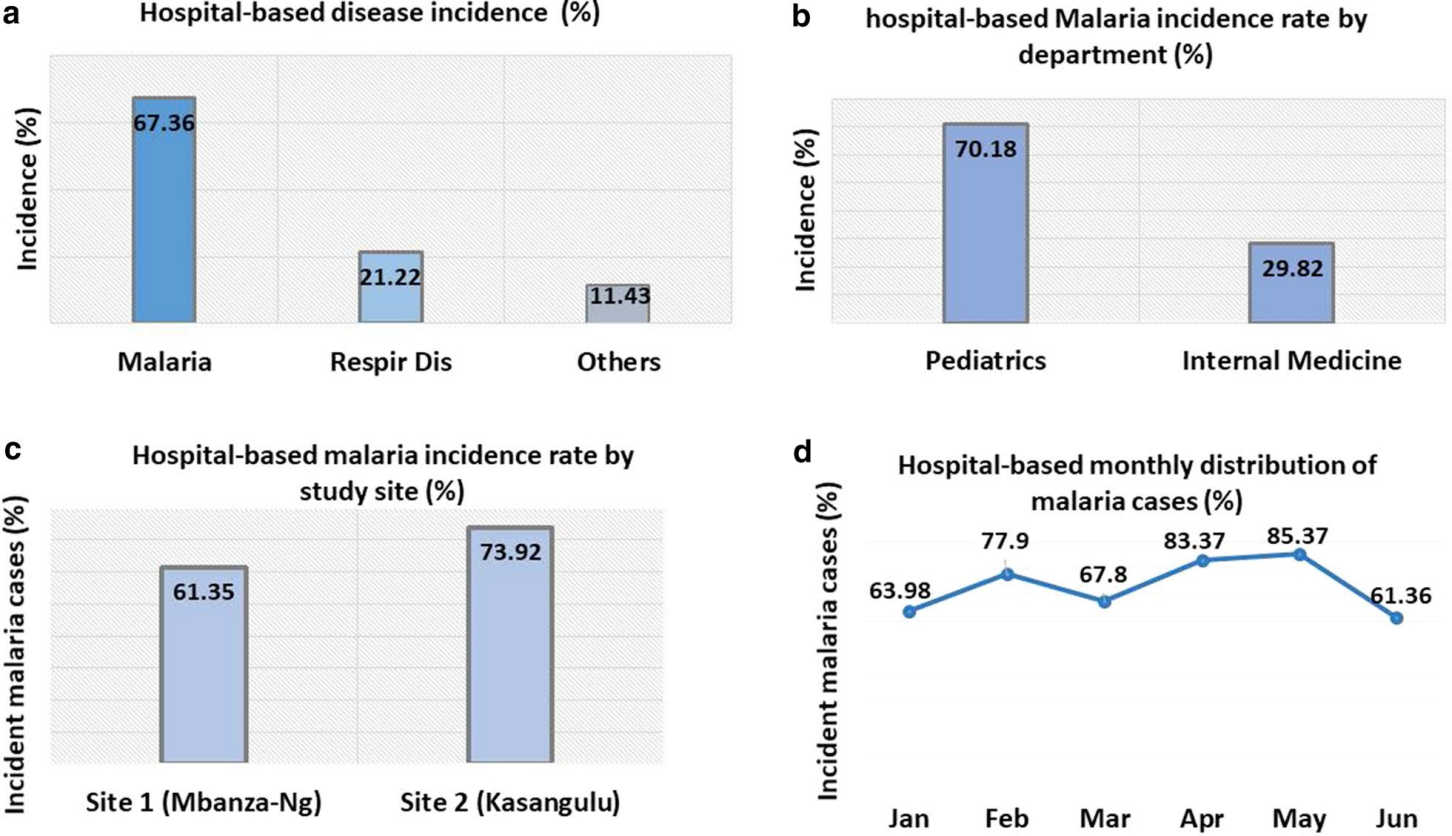
Hospital-based malaria incidence trend at referral hospitals located in study sites, Kongo Central province, DR Congo. Figure 6 shows high malaria incidence rates in the two referral health settings that participated in this study (**a**), in pediatric departments (**b**), study sites (**c**). When considering hospital-based monthly incidence rate in first semester of 2017 (**d**), malaria was more frequent in February, April and May which correspond to months with high pluviometry in western area of DR Congo

Figure 6 shows the disease distribution at the two referral hospitals located at both study sites. Overall malaria incidence was high, 67.36% (1,108/1,645), followed by respiratory diseases (21.22%; 349/1,645) and other conditions, including gastrointestinal disorders (11.43%; 188/1,645) (Fig. 6a). Pediatric malaria was more frequent, 69.67%, whereas of the disease accounted for 30.33% of patients admitted to Internal Medicine Departments (Fig. 6b).

When considering malaria status at each study site, the rate was 61.35% at Loma county (WASH site) and 73.92% in Kasangulu (Control site) (Fig. 6c). Highest malaria incidence rates were observed between April (83.37%) and May (85.37%); it decreased in June 61.36%, which corresponds to the dry (cold) season (Fig. 6d).

## Discussion

This study was carried out in two Congolese towns, Mbanza-Ngungu and Kasangulu, in Kongo central province, DRC. The trend of RDT positivity among the participants (respondents), survey-based self-reported individual/household malaria between July 2017 and 31 March 2018 and related predictors were investigated. On the other hand, hospital-based malaria incidence for patients admitted at referral health settings located at the study sites during the first semester of 2017 was also determined. Prevalence of positive RDT among the participants and self-reported household malaria incidence were significantly reduced by WASH action. Of the survey respondents (96%) who reported having suffered from at least one malaria episode, about 33% did not have access to malaria care at a health setting. Most of them relied on self-medication, which is a common practice in poor Congolese communities.

It is noteworthy to say that while RDT results revealed the effect of community-led WASH action on malaria rate, household malaria survey helped to identify geospatial disease clusters, with high risk areas having high disease frequency. For example, households whose residences were located in proximity to river, grassy area or still/stagnant water spots had higher malaria incidence than those located far from such areas. This finding provides evidence to be used in designing outdoor antimalarial interventions. As reported by Zinszer and colleagues, vegetative indices such as elevation (altitude), surface moisture, land cover are among environmental factors associated with temperature and precipitation, which influence the distribution of malaria in most malaria endemic countries in Africa [24].

Furthermore, malaria incidence was high in local referral hospitals, compared to other diseases, according to hospital-based epidemiological data, and highest incidence occurring in June. In DRC, as well as other countries of the Central African region, June is the colder month of the year. Previous studies have confirmed the relationship between climatic conditions such as temperature and rainfall on the one hand, and malaria on the other hand [25, 26].

Amidst severe economic crisis in the DRC, caused in part by the longstanding armed conflicts, and due to poverty, unemployment, lack of national health insurance and universal health care system, accessibility to quality health care is still a mere dream for many Congolese households [13]. Additionally, the issue related to low household income might explain the considerable rate (24%) of household that did not use any preventive measures against malaria. In SSA region, health inequalities have been previously reported to be influenced by socioeconomic status. For example, a study conducted in rural Kenya [27] showed highest malaria prevalence among poorest individuals.

In the present study, high self-reported incident malaria cases were observed (with two cases or more per household) in the previous 6-month period prior to implementing interventions, according to baseline survey results. Similarly, this fact was evidenced by results from the hospital-based epidemiological study that revealed high malaria incidence. On the other hand, the baseline survey also showed higher malaria incidence in households with residences in areas with highest risk for malaria transmission, whereas it significantly decreased in the WASH action site where WASH action was implemented. This outcome implies the importance of maintaining the residential environment clean and free of mosquito breeding sites, in order to reduce the risk of disease transmission through mosquito bites.

This study also showed an association between malaria and household income status, with members of household earning less than 100 $USD per month having higher malaria risk. A recent systematic review and meta-analysis conducted by Degarege et al*.* [28] also confirmed this fact. Similarly, another study by Tasting et al*.* [29], which focused on paediatric malaria, showed that the odds of malaria infection were higher in poorest children as compared with the least poor ones, suggesting that socioeconomic development could be an effective and sustainable intervention against malaria. Obviously, households with low income have difficulty to regularly afford the cost of effective anti-malaria preventive measures.

This study can be considered a valuable scientific contribution, as it provides novel insights on malaria risk distribution according to residential environment, accessibility to preventive measures and malaria care, as well as the role of community involvement in the fight against this deadly disease, particularly in malaria endemic countries.

As limitations, low literacy rate in rural areas in DRC [13] could be an obstacle in educational interventions. In addition, respondents with low literacy could have difficulty to report malaria events occurring in their household in the previous six-month period during the surveys, which might cause recall bias. Nonetheless, this study has a number of strong points. Home visits by teams of surveyors allowed to have 100% of participation rate across the study period. Furthermore, WASH intervention was implemented with the active participation of community members and could significantly reduce malaria rates. This could be a cost-effective intervention that be used to control malaria, in addition to commonly used antimalarial preventive strategies.

## Conclusion

This study explored malaria RDT positivity, incidence, and household malaria predictors in Kongo central province, DRC. In order to reduce the malaria burden in DRC, there is a need to consider the role of community involvement in the fight against malaria, particularly in terms of promotion of clean-living environment through interventions that reduce mosquito population and the risk of disease transmission. In DRC, a malaria endemic country, the use of indoor preventive measures such as LLIN and IRS might not be enough to control the disease. According to the finding, LLIN and IRS users and non-users were equally exposed to malaria due to high disease risk (numerous mosquito breeding sites, high population in the living environment). There is a necessity for DRC health policymakers to consider integrating indoor and outdoor preventive measures, with the involvement of communities for an efficient malaria control. Furthermore, DRC government should make primary health care accessible to its population in effort towards achieving universal health coverage.

## Data Availability

The datasets analyzed during this study are available from the corresponding author on reasonable request. The study data are available from the corresponding author.

## Abbreviations

HRP2: Histidine-rich protein 2
IRS: Indoor residual spraying
LLIN: Long-lasting insecticide-treated bed net
MIS: Malaria indicator survey
PHAST: Participatory hygiene and sanitation transformation
pLDH: Plasmodium lactate dehydrogenase
WASH: Water, sanitation and hygiene
